# Non-B DNA-Forming Motifs Promote Mfd-Dependent Stationary-Phase Mutagenesis in *Bacillus subtilis*

**DOI:** 10.3390/microorganisms9061284

**Published:** 2021-06-12

**Authors:** Tatiana Ermi, Carmen Vallin, Ana Gabriela Regalado García, Moises Bravo, Ismaray Fernandez Cordero, Holly Anne Martin, Mario Pedraza-Reyes, Eduardo Robleto

**Affiliations:** 1 School of Life Sciences, University of Nevada, Las Vegas, 4505 S Maryland Pkwy, Las Vegas, NV 89154, USA; tatianaermi@gmail.com (T.E.); vallincarmen@gmail.com (C.V.); moises.bravo.jr@gmail.com (M.B.); fernai1@unlv.nevada.edu (I.F.C.); holly.martin2@ucsf.edu (H.A.M.); 2 Department of Biology, Division of Natural and Exact Sciences, University of Guanajuato, P.O. Box 187, Guanajuato Gto. 36050, Mexico; ag.regaladogarcia@gmail.com (A.G.R.G.); pedrama@ugto.mx (M.P.-R.)

**Keywords:** mutagenesis, non-B DNA, hairpins, G4 DNA, *B. subtilis*, stationary phase

## Abstract

Transcription-induced mutagenic mechanisms limit genetic changes to times when expression happens and to coding DNA. It has been hypothesized that intrinsic sequences that have the potential to form alternate DNA structures, such as non-B DNA structures, influence these mechanisms. Non-B DNA structures are promoted by transcription and induce genome instability in eukaryotic cells, but their impact in bacterial genomes is less known. Here, we investigated if G4 DNA- and hairpin-forming motifs influence stationary-phase mutagenesis in *Bacillus subtilis*. We developed a system to measure the influence of non-B DNA on *B. subtilis* stationary-phase mutagenesis by deleting the wild-type *argF* at its chromosomal position and introducing IPTG-inducible *argF* alleles differing in their ability to form hairpin and G4 DNA structures into an ectopic locus. Using this system, we found that sequences predicted to form non-B DNA structures promoted mutagenesis in *B. subtilis* stationary-phase cells; such a response did not occur in growing conditions. We also found that the transcription-coupled repair factor Mfd promoted mutagenesis at these predicted structures. In summary, we showed that non-B DNA-forming motifs promote genetic instability, particularly in coding regions in stressed cells; therefore, non-B DNA structures may have a spatial and temporal mutagenic effect in bacteria. This study provides insights into mechanisms that prevent or promote mutagenesis and advances our understanding of processes underlying bacterial evolution.

## 1. Introduction

Experiments in the 1950s established that mutations are replication-dependent events that occur randomly during growth [1]. However, studies in the past few decades have uncovered transcription-dependent mutagenic mechanisms and their underlying factors. These types of mechanisms affect mutagenesis at specific genomic regions as a function of transcriptional activity [2,3,4,5,6]. One enzyme important in transcription-dependent mutagenesis is Mfd, the transcription-repair coupling factor [4,7,8]. Mfd gets recruited to stalled RNA Polymerase (RNAP) at damaged DNA sites and initiates high-fidelity transcription-coupled repair (TCR). Recent structural studies indicate that Mfd is a more dynamic protein than previously described and can function outside of TCR and in the absence of exogenous damage. Mfd was shown to bind [9] and translocate on double-stranded DNA [10] as well as to discriminate between different transcription elongation-stalling events [11]. Moreover, Mfd is proposed to be a pro-mutagenic/evolvability factor [12,13,14] that can associate with RNAP [9] and interact with different repair proteins to promote mutations [15,16]. More recently, it has been described as a factor affecting global transcription [17,18]. An exciting aspect raised by the transcription-induced mutagenesis mechanism is whether intrinsic sequences with the potential to form non-B DNA structures influence such a process in an Mfd-dependent manner.

Non-B DNA sequences can form structures that deviate from the canonical right-handed Watson and Crick structure. In vitro studies found that non-B DNA structures are promoted and stabilized by transcription [19,20] and can block the Mammalian and T7 phage RNA polymerases [21,22]. In eukaryotic cells, non-B DNA-forming sequences associate with genomic instability [23,24] as well as transcription and replication disruptions [25,26,27]. Many studies have shown that these types of events lead to recognition by and recruitment of several DNA repair factors such as CSB, the functional human homolog of Mfd, helicases, and error-prone polymerases [26,28,29]. However, we know less about the role of non-B DNA in bacteria, mainly whether it influences genome instability and the factors that promote or prevent mutagenesis at these sites.

Research on the influence of non-B DNA structures in mutagenesis in bacteria has focused on two types of sequence motifs predicted to form G-quadruplex (G4) DNA and hairpin structures [30,31]. A G4 DNA sequence has four equal guanine tracts separated by three loop regions: G_3+_N_Y1_G_3+_N_Y2_G_3+_N_Y3_G_3+_. The four guanine tracts come together to form a tetrad plane via Hoogsteen base-pairing and stack vertically, creating a four-stranded structure [32]. Hairpin sequences are formed by inverted repeats, making a paired stem and a single-stranded DNA loop region. A recent study on *E. coli* growing cells found that the presence of a G4 DNA sequence in an actively transcribed reporter gene strongly increased mutation rates. Interestingly, they found that Hfq promoted this process by binding and stabilizing the G4 structure [31]. Hfq is a regulatory nucleic acid-binding protein whose function has broadened recently [33], and now extends to interactions with alternate DNA structures and a role in *B. subtilis* stationary-phase survival [34]. Similarly, hairpin sequences can promote genetic instability through slippage events that result in insertions or deletions (indels) during replication [35]. Notably, when reporter genes containing hairpin-forming sequences were transcriptionally active, mutation frequencies increased and correlated with promoter strength [36]. These studies in *E. coli* suggest that non-B DNA structures may have a role in mutagenesis, particularly in transcribed regions, but does this occur in other bacterial species? If so, what factors, besides Hfq, promote such a mechanism? 

This study investigated if G4 DNA and hairpin-forming motifs influence stationary-phase mutagenesis (SPM) in *Bacillus subtilis*. *B. subtilis* is a Gram-positive sporulating bacterium adapted to feast and famine cycles in the soil’s upper layers [37]. Upon the onset of stress, such as starvation, *B. subtilis* cells activate gene expression programs, enter a non-growing state, and differentiate into distinct subpopulations [38]. Our previous observations have demonstrated that increased mutagenesis occurs in highly transcribed regions [39] and in distinct cell subpopulations [40,41]. Mfd also acts in coordination with nucleotide excision repair (NER), base excision repair (BER), and the transcription factor GreA to promote mutagenesis during stationary phase [15,42]. These observations make *B. subtilis* an attractive model to test our hypothesis that non-B DNA-forming motifs influence Mfd-dependent stationary-phase mutagenesis at transcribed regions. 

**Figure 1:**
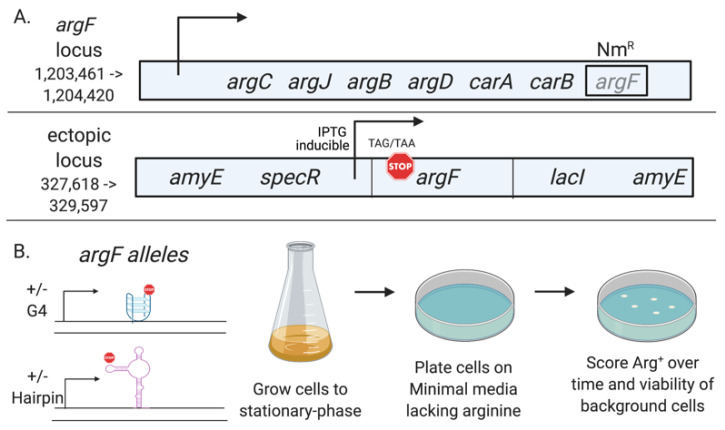
Schematic of the stationary-phase assay. (**A**) The *argF* gene was replaced with a selectable neomycin marker at its chromosomal locus. Then, IPTG-inducible *argF* alleles containing nonsense codons and differing in their potential to form non-B DNA structures were introduced into an ectopic chromosomal region. (**B**) Stationary-phase mutagenesis assay. Cells are grown to stationary phase and plated on minimal media lacking arginine. Then, we scored Arg^+^ mutants over time.

We developed a system to measure non-B DNA’s influence on *B. subtilis* stationary-phase mutagenesis by deleting the wild-type *argF* at its chromosomal position and introducing IPTG-inducible *argF* alleles differing in their ability to form hairpin and G4 DNA structures into the *amyE* locus. To design these *argF* alleles, we used in silico tools to find endogenous non-B DNA sequences in the *argF gene* and introduced point mutations to the open reading frame to either increase or decrease their predicted structure stability. We then introduced a nonsense codon in a region corresponding to a loop in the structure, which resulted in a non-functional truncated ArgF (confers auxotrophy) and a system to measure reversions to Arg^+^ (Figure 1). Using this system, we found that sequences predicted to form non-B DNA structures promoted mutagenesis in stationary-phase cells; such a response did not occur in growing conditions. We also showed that Mfd promotes the formation of mutations within the predicted structures. 

## 2. Materials and Methods

### 2.1. Strain Construction

The bacterial strains used in this study are shown in Table 1. For detailed strain construction, see supplemental material Non-B DNA strain construction. Briefly, Mfold [43] and QGRS mapper [44] were used to find sequences with the potential to form hairpin and G4 DNA structures, respectively, in the *argF* ORF. Once the sequences were found, synonymous point mutations were introduced into positions that were predicted to strengthen or destabilize the structure. A nonsense codon was then engineered into a region of the structure corresponding to a loop. For the +/−hairpin strains, we introduced an ochre nonsense codon at position 37Q (CAA→TAA) corresponding to the loop part of the predicted hairpin before disruption. For the +/−G4 strains, we introduced an amber nonsense codon at position 70Q (CAG→TAG) corresponding to the second loop of the predicted G4 structure. This nonsense codon resulted in a truncated ArgF protein and arginine auxotrophy. The new defective *argF* alleles were then cloned into pDR111 downstream of the IPTG-inducible *Pspac* promoter, transformed, and recombined into the *amyE* locus of the *argF* deletion strain CV4000 (see Figure 1). The Mfd^−^ strains were constructed by transforming genomic DNA from YB9801 [8] into the +/−hairpin and +/−G4 strains and selecting on TBAB plates containing tetracycline.

### 2.2. Stationary-Phase Mutagenesis Assay

We performed a stationary-phase mutagenesis assay on each strain [47]. Strains were streaked out on TBAB plates with the appropriate antibiotic and incubated overnight at 37 °C. The next day, a colony from the TBAB plate was used to inoculate 2 mL of Penassay Broth (PAB). The cells were grown overnight at 37 °C with aeration. An aliquot of the overnight was then added to a 250 mL Erlenmeyer culture flask containing 10 mL of PAB medium and 10 μL of 1000× Ho-Le trace elements. Cells were grown at 37 °C with aeration (250 rpm) to stationary phase. Growth was monitored with a spectrophotometer measuring O.D. at 600 nm (OD_600_). Cells were harvested 90 min after cessation of exponential growth and centrifuged at 4000 rpm for 5 min, the supernatant was decanted, and the cells were resuspended in Spizizen minimal salts (SMS); this process was repeated twice. To determine initial cell titers, we serial diluted and spread plated 100 μL of cells on minimal media supplemented with 100 μg/mL arginine. Colonies were counted after 48 h of incubation at 37 °C. Then, 100 μL aliquots were plated on minimal media containing 0.1 mM IPTG and no arginine (*n* = 5). The plates were incubated for nine days at 37 °C. Each day plates were scored for the appearance of Arg^+^ colonies. The initial titers were used to normalize the cumulative number of revertants per day to the number of the CFU plated. To assay the background survival of cells, the viability was tracked by taking agar plugs every other day, serial diluting, and plating on minima media supplemented with 100 μg/mL arginine.

### 2.3. Arg^+^ Mutant Analysis

To characterize Arg^+^ mutants, we performed a tRNA suppressor analysis and DNA sequencing [47]. Suppressor analysis was conducted by first patching between 20 and 25 Arg^+^ colonies collected from days 5–9 of each replicate trial onto the same media used in the SPM experiment (minimal media lacking arginine supplemented with 0.1 mM IPTG) to eliminate transient Arg^+^ prototrophs. Previous studies determined that analyzing mutants that arose after day 5 would avoid selecting slow-growing mutants from growth [47]. Arg^+^ mutants that grew were stocked and then patched on minimal media lacking either methionine or histidine. Our background *B. subtilis* YB955 strain is also auxotrophic for methionine and histidine due to ochre and amber nonsense codon in the *metB* and *hisC* genes, respectively [47]. Growth of the Arg^+^ mutants on either trace met and trace his plates was scored after 24 h and 48 h. If growth occurred, those revertants were considered tRNA suppressors. For those Arg^+^ mutants determined not to be tRNA suppressors, we performed a colony PCR amplifying and sequencing the complete *argF* gene.

### 2.4. Fluctuation Test

We conducted a fluctuation test to determine the mutation rates for arginine prototrophy during growth for each strain. Briefly, an overnight aerated culture grown overnight in 2 mL of PAB at 37 °C were diluted 1:10 in 1× Spizizen minimal salts (SMS), vortexed, and diluted 1:10 again in this solution. Aliquots of 0.5 mL of the second 1× SMS solution were added to 49.5 mL of PAB supplemented with 0.1 mM IPTG. The cells were vortexed, and 1 mL of the 10^−4^ mixture was dispensed into 40 different 18 mm test tubes. Cells were grown for 12 h at 37 °C with aeration (250 rpm). Each test tube culture was pelleted, washed, resuspended in 100 μL of 1× SMS, and individually plated on minimal media lacking arginine with 0.1 mM IPTG. In addition, three individual test tubes were serial diluted and plated on minimal media supplemented with 100 μg/mL arginine to determine cell count. Plates were incubated at 37 °C for 48 h, and then colonies were counted. The mutation rates were determined using the MSS maximum likelihood method [48,49]. 

### 2.5. Statistical Analysis

To determined what type of significance test to use, we first checked the data for normality and variance using a Shapiro–Wilk test and Brown–Forsythe test, respectively. Significance was determined by performing a Student’s *t*-test to compare two strains or a one-way ANOVA with a post-hoc Tukey’s test to compare more than two strains. 

### 2.6. Protein Alignment Analysis

The *B. subtilis* 319 amino acid ArgF protein sequence was inputted into pBLAST as the query sequence and blasted into the UniProtKB/Swiss-Prot (SwissProt) database. The output identified 100 ArgF amino acid sequences. The sequences were exported and aligned. Then, we analyzed position 37 and 70 using Weblogo. Weblogo reported the conservation at that site. In addition, Weblogo reported the frequency of each amino acid found at that site within the 100 ArgF sequences.

## 3. Results

### 3.1. Non-B DNA-Forming Motifs Promote Mutagenesis

#### 3.1.1. Hairpins and G4 DNA-Forming Motifs Accumulate Mutations in Stationary Phase *B. subtilis* Cells

**Figure 2:**
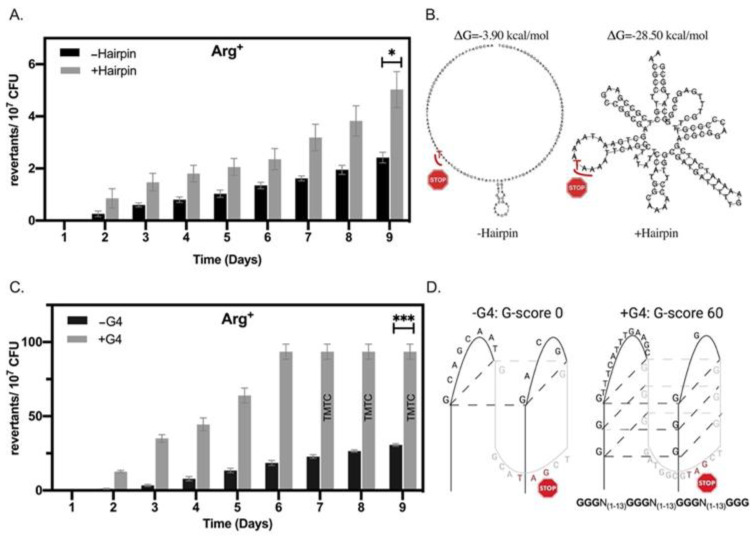
Hairpins and G4 DNA-forming motifs promote mutations in stationary phase *B. subtilis* cells. (**A**) Stationary-phase mutagenesis assay results for +/−hairpin strains. (**B**) Mfold predicted hairpin structures for 120 bp region in *argF* (positions 82–202). (**C**) Stationary-phase mutagenesis assay results for +/−G4 strains. (**D**) Schematic of predicted G4 DNA structures and G-score in *argF* (position 182–219). Please note the differences in scale between the two graphs. Bars represent the mean, and error bars are SEM (*n* = 3). Statistical significance was determined using a Student’s *t*-test * *p* < 0.05, *** *p* < 0.001.

Our stationary-phase mutagenesis assay revealed that the strains containing *argF* alleles with sequences predicted to form non-B DNA structures accumulated significantly more mutants over nine days than those *argF* alleles less likely to form such structures (Figure 2). Specifically, by day 6, the +G4 strain containing the sequence G_3_N_(1–13)_G_3_N_(1–13)_G_3_N_(1–13)_G_3_ accumulated ~3 times more mutants than the −G4, which was not predicted to form any G4 structure. Arg^+^ accumulation for the +G4 strain continued after day 6, but the number of revertants became too numerous to count compared to what was observed in the −G4. The +hairpin (120 b) strain showed a significant increase (~two fold) in the accumulation of Arg+ colonies compared to the −hairpin. Since window size is an important parameter for predicting hairpin structures [36], our approach included disrupting a hairpin structure using a smaller window size in the same region of *argF.* We found mutagenesis of the −hairpin (40 b) strain indistinguishable from the W.T. in our experiments (Supplemental Figure S6). In addition, mutagenic differences observed in all the strains were not the result of viability differences during the experiment (Supplementary Figure S7). Interestingly, the strains differing in G4 stability had more mutants over time compared to the strains differing in hairpin stability. These results are consistent with our previous report [47] and we attribute this difference to the type of nonsense codon used in the different constructs (see Supplemental TAG vs. TAA section).

#### 3.1.2. Analysis of the Arg^+^ Population

**Figure 3:**
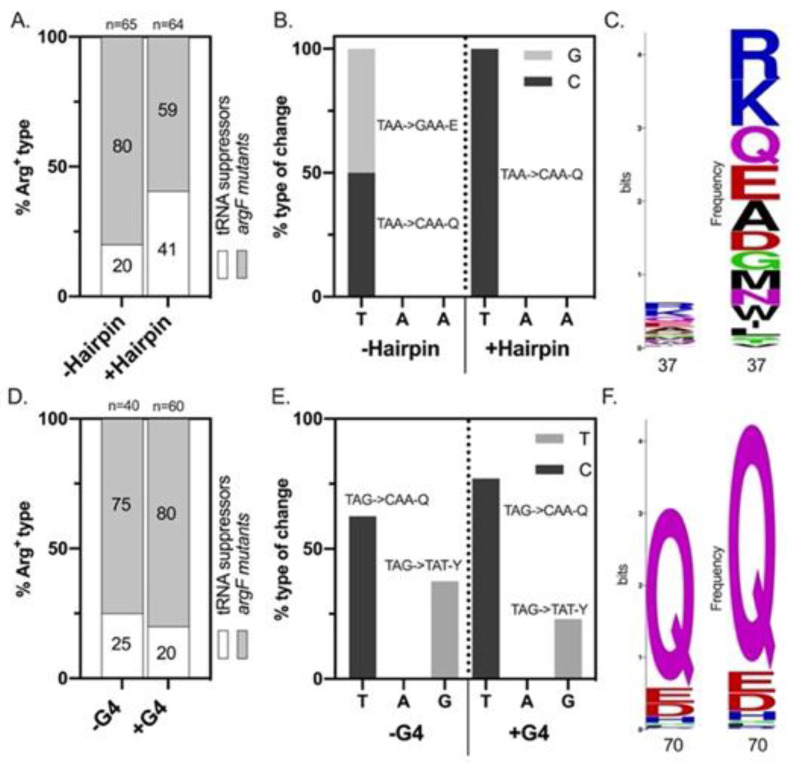
Arg^+^ revertant analysis. (**A**–**C**) +/−Hairpin results; (**D**–**F**) +/−G4 results. (**A**,**D**) The proportion of tRNA suppressors from Arg^+^ population sampled that arose from day 5–9. (**B**,**E**) argF sequencing results from Arg^+^ mutants that were determined not to be tRNA suppressors. (**C**,**F**) Protein alignment analysis of positions containing the nonsense codons 37 and 70.

Analysis of a sample of the Arg^+^ colonies that arose after day 5 indicated that two categories of mutants restored arginine prototrophy in the tested strains: (1) nonsense suppressor tRNAs and (2) mutations in *argF* (Figure 3A,D). Nonsense tRNA suppressors represented <50% of the Arg^+^ colonies sampled. Sequencing of non-tRNA suppressor mutants showed that mutations in *argF* were limited to base substitutions that changed the stop codon’s first or third position (Figure 3B,E). We did not observe deletion events in our system.

In the strains differing in hairpin stability with the ochre nonsense codon TAA at position 37, transitions in the first position from T⟶C restored the W.T. polar glutamine-Q codon, and T⟶G transversions resulted in the acidic glutamate-E codon (Figure 3B). A protein analysis that looked at 100 ArgF sequences across different phyla confirmed that this codon position had some flexibility and that glutamate-E was indeed found at this position in some bacterial species (Figure 3C). Only transitions in the first positions were observed in the +hairpin strain, and no mutations occurred in the second or third position in either of the hairpin strains. Interestingly, the proportion of tRNA suppressor mutants appeared higher in our sample of the +hairpin than in the −hairpin one (Figure 3A).

In the strains differing in G4 stability carrying the amber nonsense codon TAG at position 70, revertants displayed T→C transitions in the first codon position, which restored the W.T. polar glutamine-Q triplet, and G→T transversions in the third codon position, which resulted in the aromatic amino acid tyrosine-Y triplet (Figure 3E). Protein analysis of this codon position found that it exhibited some flexibility (Figure 3F). Additionally, glutamate-E was the second most frequent amino acid found at this position in other ArgF proteins, while tyrosine was not observed in our sample of 100 ArgF proteins. (Figure 3F). Moreover, the proportions of the revertants with these genetic changes were similar in both strains (Figure 3E). The proportion of tRNA suppressors was similar in our sample of the Arg^+^ population between the +/−G4 strains (Figure 3D). 

### 3.2. Hairpin- and G4 DNA-Forming Motifs Do Not Influence Mutation Rates in B. subtilis

**Figure 4:**
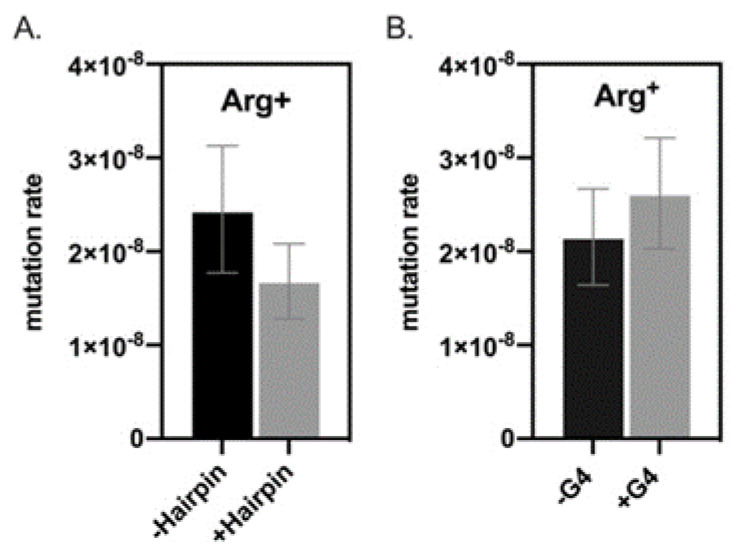
Hairpin (**A**) and G4 DNA (**B**) forming motifs did not affect mutation rates in growing *B. subtilis* cells. Bars represent mutation rates using the MSS maximum likelihood method, and error bars are confidence limits (CL_95_).

To test whether the results observed were exclusive to stationary-phase conditions, we performed a fluctuation test on the strains employed in this study. We found that the +/−hairpin and +/−G4 DNA’s predicted stability did not correlate with mutation rates in *B. subtilis* growing cells. Additionally, we did not observe differences between the sets of strains as we did in stationary phase (hairpin vs. G4) (Figure 4). This result suggests that cell physiology influences the role non-B DNA structures have in mutagenesis.

### 3.3. Mfd Promotes Mutations at Non-B DNA Sequences 

#### 3.3.1. Accumulation of Arg^+^ Mutations at Non-B DNA Sequences Was Decreased in the Absence of Mfd

**Figure 5:**
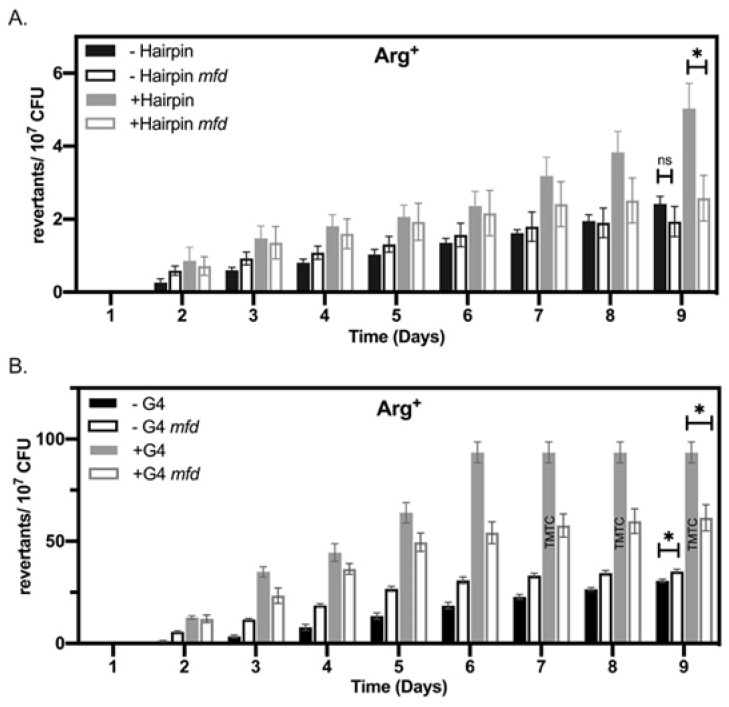
Mfd promotes mutations at hairpin (**A**) and G4 DNA (**B**) forming sequences. Bars represent the mean of at least three SPM trials, and error bars are SEM. Please note the differences in scales on the Y axis between graphs. Significance was determined using a Student’s *t*-test * *p* < 0.05.

We tested if the transcription-repair coupling factor Mfd influenced SPM at non-B DNA sequences in *B. subtilis*. We transformed the *mfd* mutation into the strains carrying *argF* alleles differing in their predicted ability to form either hairpin or G4 DNA structures and performed a stationary-phase mutagenesis assay. Disrupting *mfd* significantly decreased the accumulation of mutations by ~two-fold in the +hairpin and +G4 strains (Figure 5). This response was not observed in the −hairpin, and there was a small but significant increase in the accumulation of Arg^+^ mutants in the −G4 strain deficient in Mfd (Figure 5B). These results were not due to differences in viability (Supplementary Figure S8). 

#### 3.3.2. Analysis of the Arg^+^ Population in Mfd^−^ Cells

**Figure 6:**
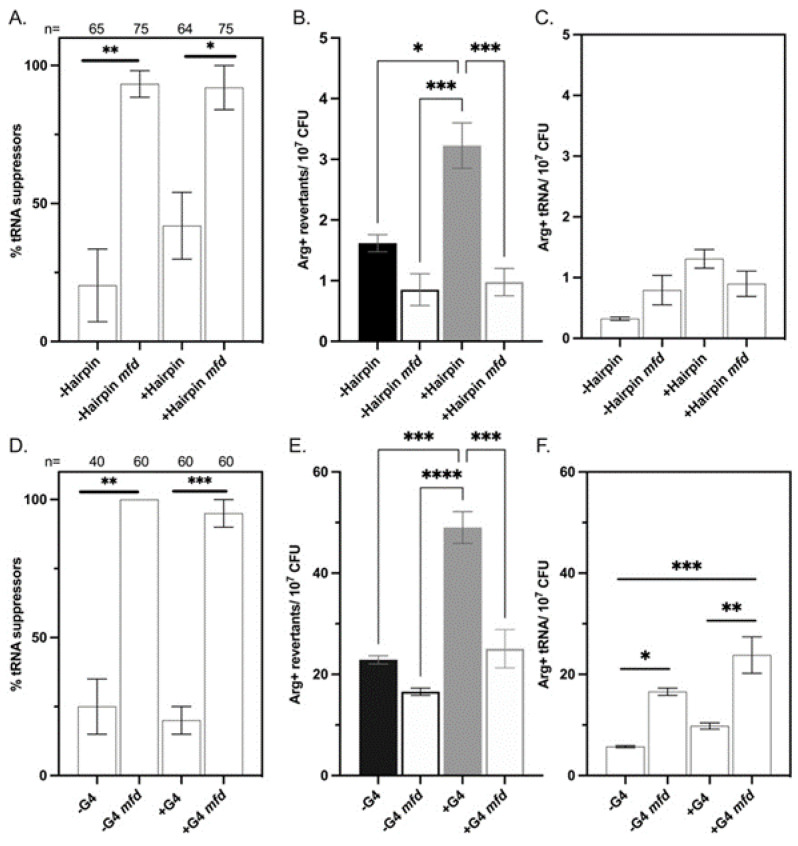
Mfd promotes mutagenesis at *argF*. (**A**–**C**) +/−Hairpin results; (**D**–**F**) +/−G4 results. (**A**,**D**) The proportion of tRNA suppressors from Arg^+^ population sampled that arose from day 5–9. (**B**,**E**) Arg^+^ mutants that accumulated from day 5–9. (**C**,**F**) tRNA suppressors that arose in the Arg^+^ population after day 5. Bars represent the mean with SEM. Significance was determined using ANOVA; * denotes *p* < 0.05, ** denotes *p* < 0.01, *** denotes *p* < 0.001, and **** denotes *p* < 0.0001.

Subsequent analysis of the Arg^+^ mutants found that Mfd deficiency abolished mutations in *argF* as almost 100% of the mutants sampled were nonsense tRNA suppressors (Figure 6A,D). Thus, the spectrum of mutations changed drastically in the Mfd deficient strains. We estimated the total number of tRNA suppressors in this Arg^+^ population based on the sampling of mutants that arose at days 5–9 during the SPM trials. This analysis suggested that nonsense tRNA suppressors significantly increase in the +/−G4 lacking but not in the +/−hairpin strains in the Mfd^−^ background (Figure 6C,F). The genetic changes that confer arginine prototrophy in *argF* (Figure 3E) suggest that mutagenesis of tyrosine (first position in the G/ATA anticodon) and glutamine (third position in the TTG anticodon) tRNA genes are increased in the absence of Mfd.

## 4. Discussion

### 4.1. An In Vivo System to Measure the Effects of Non-B DNA-Forming Motifs on Bacterial Mutagenesis

We investigated if non-B DNA-forming motifs, such as hairpins and G4 DNA sequences, contribute to stationary-phase mutagenesis in *B. subtilis*. First, we exploited the fact that this bacterium does not have a strong codon bias [50,51,52] to develop a system that altered endogenous non-B DNA sequences in the *argF* gene by introducing point mutations that increase or decrease their predicted stability. We then tested the effect of these changes on mutagenesis during growth and stationary phase.

Our approach to construct the non-B DNA sequence variants included the use of in silico tools that predict their stability based on extensive thermodynamic studies. We used Mfold [43] and QGRS mapper [44] to measure hairpins and G4 DNA stability, respectively. Previous in vitro [21] and in vivo studies [31] used optimized sequence motifs in tandem arrangements. These arrangements are not commonly found in coding regions, likely due to functional constraints. In contrast, we worked with an endogenous system by altering sequence motifs within a coding region. The G4 motif used in our experiments had longer asymmetrical loops (G_3_N_(1–13)_G_3_N_(1–13)_G_3_N_(1–13)_G_3_) than those commonly used (G_3_N_(7)_G_3_N_(7)_G_3_N_(7)_G_3_), and thermodynamic studies have shown that sequences having asymmetrical loops can still form stable G-quadruplex structures [24,53]. Additionally, G4 DNA containing a single-nucleotide loop, like the one used in this study, was shown to be thermally stable and to incite genomic instability in eukaryotic genomes [24]. In this regard, we performed a G4 motif search across the *B. subtilis* genome and found that the canonical G4 motif occurred four times in coding regions, while the G4 motif used here appeared in 39 genes (Supplemental Table S2). Further characterization of these genes showed they were distributed throughout the *B. subtilis* chromosome and many coded for membrane-bound proteins (Supplemental Figures S9 and S10).

To design the constructs that tested the effect of hairpins within coding regions on SPM, we used two window sizes because this parameter was reported to affect their stability, particularly in the context of their transcription [36]. High levels of transcription generate more supercoiling, which increases the likelihood of forming single-stranded DNA and intrastrand base pairing between distant DNA residues [54,55]. We found that disrupting a hairpin using a 40 b window size did not affect mutation levels (Supplemental Figure S6); in contrast, using a 120 b window size did. The IPTG-inducible Pspac promoter used in our study is robust, suggesting that the design using a larger window size provided a better parameter for forming single-stranded hairpins. Studies have used window sizes ranging from 30–300 bases [25,36]. In summary, the features we used to design the non-B DNA forming sequences are consistent with what is observed within gene coding regions and account for conditions in which a region is robustly transcribed.

### 4.2. Non-B DNA-Forming Motifs in Transcribed Coding Regions Promote Mutations in Nutritionally Stressed Cells

Early in silico analysis in bacteria found that non-B DNA forming sequences were enriched in noncoding regions, including promoters and untranslated regions (UTR). This finding led researchers to focus on elucidating the role of these structures in gene regulation [56,57]. Indeed, a study in a polyextremophile, *Deinococcus radiodurans*, which contains G4-motifs in promoter regions, found that adding a G4 stabilizing ligand led to repression of DNA repair genes and decreased radioresistance [58].

The effect of DNA structures found in bacterial regulatory regions continues to be well studied [59]. However, very little is known about what happens to alternate DNA structures that are within an ORF. Wright and colleagues proposed that hairpin structures formed during transcription in coding regions were hotspots for mutagenesis in *E. coli* [30]. More recently, a study examined the role of G4 structures in genome instability in *E. coli*-transcribed regions and found that these sequences increased the rate of deletions [31]. Here, we investigated if G4 DNA- and hairpin-forming motifs as intrinsic elements that influence stationary-phase mutagenesis in *B. subtilis*. We found that non-B DNA forming sequences promoted mutagenesis in stationary-phase cells. Specifically, sequences increased or decreased in the likelihood to form a hairpin, and G4 DNA were increased and decreased in the accumulation of Arg^+^ mutants, respectively. Furthermore, unlike results in *E. coli*, this response was only observed in nutritionally stressed cells and did not occur in growing cells. This result underlines the contribution of mutagenic processes taking place in non-replicating conditions to the evolutionary process and the importance of using different model systems. Moreover, this work provides support to the concept that non-B DNA plays a role in bacterial transcription-dependent mutagenic processes.

### 4.3. Mfd Promotes Mutations at Non-B DNA Sequences

To identify factors governing the process by which non-B DNA-forming motifs promote the accumulation of mutations, we investigated if the transcription-repair coupling factor Mfd promoted mutations at non-B DNA sites. Mfd is a factor shown to promote transcription-dependent mutagenesis and is recruited to genomic regions containing non-B-DNA-forming motifs [17,28]. Disrupting *mfd* led to significant decreases in mutagenesis levels in the +hairpin and +G4 DNA strains. Subsequent analysis of the Arg^+^ population that arose in the absence of Mfd showed that the vast majority of revertants were tRNA suppressors in all strains; *argF* mutants were abolished in the absence of Mfd. This result is consistent with our previous studies in stationary-phase mutagenesis in *B. subtilis* [8,60]. This result further supports the promutagenic role of Mfd at transcribed regions and suggests that non-B DNA sites in bacteria influence Mfd-dependent mutagenesis.

Mfd associates with RNAP when transcription elongation is blocked by a bulky DNA lesion. Upon contacting the blocked RNAP, Mfd undergoes a series of conformational changes that recruit nucleotide excision repair proteins [11]. However, recent single-molecule resolution and structural studies suggest that Mfd and RNAP interact, even in the absence of DNA damage, and that Mfd works to modulate transcription [9,10]. In this context, in vivo and in vitro reports showed that non-B DNA structures formed in the DNA or RNA could interrupt transcription, especially when they are found in the coding strand [21,61,62,63]. In *B. subtilis*, loss of Mfd led to increases in RNAP association at genes enriched with hairpins [17]. Similarly, deficiencies in CSB, the functional homolog of Mfd, caused RNAPII pausing at G4 DNA motifs in a neuroblastoma cell line [28]. Considering these observations, one can propose a model in which transcription promotes the formation of alternate structures, through changes in supercoiling, in the DNA or mRNA with the potential to impede transcription. We speculate that the stability of such structures defines whether the impediment is resolved by Mfd through transcription rescue (low structure stability) or disassembly of the elongation complex (high structure stability) and recruitment of error-prone repair of DNA. In *E. coli*, Mfd promotes mutagenesis in stressed cells via the formation of R-loops and recombination intermediates that generate point mutations or amplifications in a Lac^+^ mutagenesis system [4]. This system requires recombination functions and activation of the sigma S regulon (amplification) and the DinB (point mutations) [64]. Unlike in *E. coli*, Mfd-dependent mutagenesis in *B. subtilis* operates independently of recombination functions [47] and can operate by interacting with components of the NER and BER systems [15,16,65]. It is also possible that the loss of Mfd causes depression in mutagenesis indirectly. A recent RNAseq study showed profound changes in the transcriptome of Mfd^−^ cells compared to wild-type stationary-phase cells. Almost half the genes in the *B. subtilis* genome were dysregulated, including factors previously identified to promote SPM, such as GreA [42], PolY [66], PolX [67], and others [15]. Therefore, it is possible that the Mfd effects observed in mutagenesis are the indirect product of dysregulation of a factor(s), such as a helicase or error-prone polymerase involved in mutagenesis at non-B DNA forming sequences. Interestingly, a previous study identified Hfq as a factor that promotes mutation at G4-DNA motifs in *E. coli* growing cells [31]. Like Mfd, the role of Hfq has been broadening [33], and like Mfd, cells deficient in Hfq have transcriptome changes and decreased survival during stress in *B. subtilis* [34]. Future work will determine if Hfq, along with Mfd, contribute to stationary-phase mutagenesis at non-B DNA motifs.

## 5. Conclusions

Non-B DNA sequences are present in all domains of life. These structures have a role in genome instability and disease in humans, but we know less about their function in bacteria. Here, we showed that non-B DNA-forming motifs promote genetic variation in *B. subtilis*, particularly in transcribed coding regions in stressed cells in an Mfd-dependent manner. Future work studying SPM in *B. subtilis* will discern between the competing but not necessarily exclusive hypotheses that Mfd works directly or indirectly to promote the formation of mutations at non-B DNA sequences in actively transcribed genes. This type of study provides insight into transcription-induced mutagenic mechanisms limiting mutations to times of stress and defined genomic regions, and it improves our view of evolution.

## Figures and Tables

**Table 1:** Strains and plasmids used in this study.

Strain or Plasmid	Relevant Genotype	Source or Reference
CV1000 (+Hairpin)	*metB5, hisC952, leuC427, argF::neo,* *amyE::pHS− argF+SLS*	This study
CV2000 (−Hairpin)	*metB5, hisC952, leuC427, argF::neo,* *amyE::pHS− argF−SLS*	This study
CV1001 (+Hairpin Mfd^−^)	*metB5, hisC952, leuC427, argF::neo, mfd::tc,* *amyE::pHS− argF+SLS*	This study
CV2009 (−Hairpin Mfd^−^)	*metB5, hisC952, leuC427, argF::neo, mfd::tc,* *amyE::pHS− argF−SLS*	This study
TE300 (+G4)	*metB5, hisC952, leuC427, argF::neo,* *amyE::pHS− argF+G4*	This study
TE302 (−G4)	*metB5, hisC952, leuC427, argF::neo,* *amyE::pHS− argF−G4*	This study
TE400 (+G4 Mfd^−^)	*metB5, hisC952, leuC427, argF::neo, mfd::tc* *amyE::pHS− argF+G4*	This study
YB9801 (Mfd^−^)	*metB5, hisC952, leuC427, mfd::tet*	[8]
CV4000 (*argF-)*	*metB5, hisC952, leuC427, argF::neo*	[45]
TE402 (−G4 Mfd^−^)	*metB5, hisC952, leuC427, argF::neo, mfd::tc* *amyE::pHS−argF−G4*	This study
pBEST502	neomycin resistance (Nm^r^) gene	[46]
pDR111	*pdr111 amyE-hyper-SPANK (spec)*	Rudner lab

## Data Availability

Data is contained within the article or supplementary material.

## Supplementary Materials

The following are available online at https://www.mdpi.com/article/10.3390/microorganisms9061284/s1, Figure S1: Prediction of hairpin structures across the *argF* gene using a sliding window analysis and in silico tools. Figure S2: Hairpin-forming motifs and predicted structures of the WT and +/−Hairpin (120) strains. Figure S3: Hairpin-forming motif and predicted structure of WT and −Hairpin (40) strains. Figure S4: G4-forming motif and predicted structure of +G4 and −G4 strains. Figure S5: Codon analysis using the Kazusa Codon Usage Database of regions in *argF* that were changed to disrupt or stabilize an endogenous non-B DNA structure. Figure S6: The −Hairpin (40bp strain) is indistinguishable from the W.T. Figure S7: Viability of the non-revertant population for the non-B DNA strains during the SPM assay. Figure S8: Viability of the non-revertant population for the non-B DNA strains and their Mfd deficient counterpart during the SPM assay. Figure S9: A. Schematic showing the chromosomal distribution of the 39 genes that contained the G4 motif used in this study. Figure S10: Modified figure from Bsucyc.org showing the cellular function of the 39 genes that contained the G4 motif used in this study. Table S1: QGRS Pattern search in argF ORF. Table S2: QGRS Pattern search in *B. subtilis* coding regions. Table S3: QGRS Results. Table S4: Primers used in this study. Table S5: Summary of Phenotype Test.
