# Supplementary material for: Non-B DNA-Forming Motifs Promote Mfd-Dependent Stationary-Phase Mutagenesis in Bacillus subtilis
Source: Microorganisms. 2021 Jun 12;9(6):1284. doi: 10.3390/microorganisms9061284 (PMC8231525; doi:10.3390/microorganisms9061284)
Supplement: Supplementary file 1 [file microorganisms-09-01284-s001.zip › microorganisms-1239100-supplementary.pdf]

Supplementary Material

2.1. Non-B DNA strain construction

Generally, arginine biosynthesis in bacteria begins with glutamate and takes eight steps to complete [1]. The a*rgF* gene codes for an ornithine carbamoyl-transferase enzyme that converts L-ornithine and carbamoyl-phosphate into L-citrulline, this reaction is the 6th step in the L-arginine biosynthesis II pathway in *B. subtilis* [1][2]. The genes that carry out arginine biosynthesis in *B. subtilis* are divided into three operons across the chromosome and *argF* is the last gene in an operon that contains: *argCJBD-carAB-argF*. This operon is repressed by the AhrC repressor when arginine is available [3]. Mutations in *argF and argH* have been shown to render *B. subtilis* cells auxotrophic for arginine, a property that we have previously used to develop a plate-selection assay [4]. Arg+ suppressor mutations in the Δ*argF* deleted background, and in the empty vector control, were undetectable during growth and stationary phase, and restoration of the *argF* deletion strain confers a WT phenotype. These results showed that the *argF* was an appropriate gene marker for our studies.

2.1.1 Design of the +/- Hairpin strains

To generate *argF* alleles containing hairpin-forming sequences with different stabilities, we used Mfold [5] and Vienna [6] web tools in the DNA folding setting. These tools predict hairpin structures in a size-adjustable nucleic acid sequence and consider parameters such as base stacking interactions and mismatches to calculate a Gibbs free energy stability score. We selected two sliding window sizes (40 b and 120 b), with a step size of 1 nucleotide, to find hairpin rich regions in the *argF* open reading frame (ORF). The selected window sizes fall within the range of previously used window sizes to search for hairpins that form in conditions of low (40 b) and high (120 b) levels of transcription [7][8][9]. The IPTG-inducible promoter in our studies is robust, and we expected the larger window to be a better predictor for hairpin formation and to influence mutagenesis. We included the smaller 40 b window in our analysis as a control. Our analysis found a hairpin rich region in the first 300 b of the gene, consistent with a Dinamelt [10] prediction of hairpins across the gene (Figure 1).

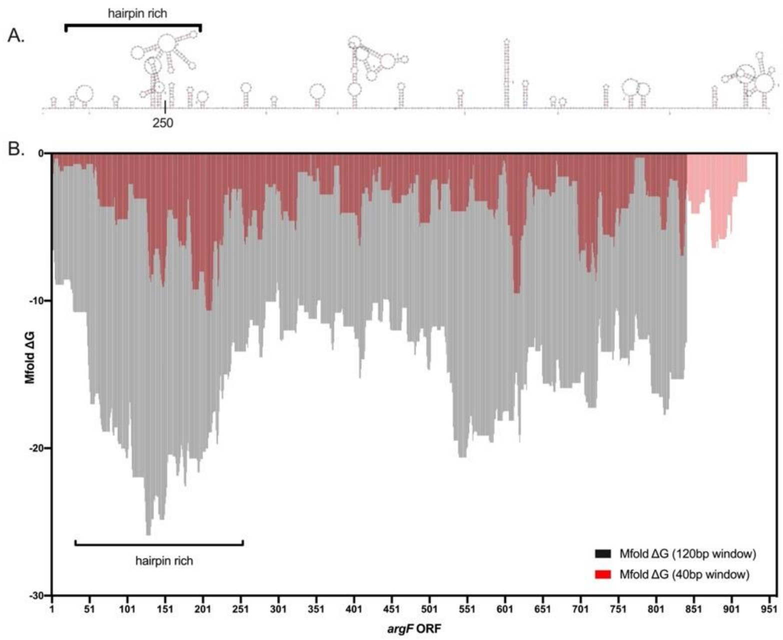

Figure S1. Prediction of hairpin structures across the *argF* gene using a sliding window analysis and in silico tools. A. Dinamelt prediction of hairpins across the *argF* gene using default settings. B. Histogram that shows the results of a sliding window analysis. The *argF* gene was split into window sizes of 40bp and 120bp with a step of 1bp, and then Mfold was used to predict the hairpin stability of every window size generated. The Mfold stability reported as a Gibbs free energy score was plotted across the *argF* ORF.

We chose two sequences within this hairpin rich region for the 40 b- and 120 b-analyses to introduce synonymous base pair changes and alter the stability of the predicted hairpin. The introduced changes were targeted to remove or add base-pairing within the stem portion of the hairpin. Of note, the hairpin predicted to form in the 40bp window falls within the 120bp window hairpin. To create the -Hairpin (120bp) strain we introduced 26 synonymous point mutations into the WT sequence to disrupt base pairing in the predicted structure. Analysis with Mfold and Vienna confirmed the changes decreased the predicted stability of the structure (Figure S2). To create the +Hairpin (120bp) strain we introduced six synonymous point mutations into the WT sequence to increase base pairing in the predicted structure. Mfold and Vienna confirmed the changes increased the predicted stability of the structure (Figure S2). To create the -Hairpin (40bp) control strain, we introduced 4-point mutations into the WT sequence to disrupt base pairing in the predicted structure (Figure S3).

	WT	+Hairpin (120)	-Hairpin (120)
Mfold	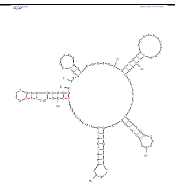	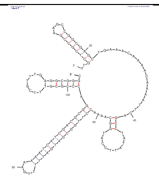	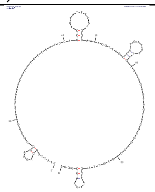
ΔG	-19.14 kcal/mol	-28.98 kcal/mol	-4.61 kcal/mol
Vienna	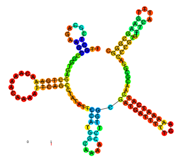	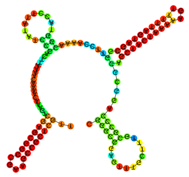	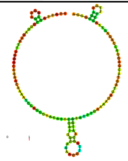
ΔG	-17.80 kcal/mol	-28.50 kcal/mol	-3.90 kcal/mol

	Sequence
**WT**	aac gcc TTG CTC GCA GAA GCC GGT GAG CTG AAA CAA AAC AAA ATT CAG CCT ATA TTC CAT GGC AAA ACC TTG GCG ATG ATT TTT GAA AAA TCA ACG CGG ACC CGT GTT TCA TTT GAA GCG GGC atg gcg
**-Hairpin (120)**	aac gca TTA GCA GAA GCC GGA GAA TTA AAA CAA AAC AAA ATC CAG CCT ATA TTT CAT GGC AAA ACG TTA GCA ATG ATA TTT GAA AAG AGT ACG AGG ACC AGA GTT AGC TTT GAA GCG GGA atg gct

**+Hairpin (120)**	aac gcc TTG CTC GCC GAA GCC GGC GAG CTG AAA CAA AAC AAA ATT CAG CCT ATA TTC CAT GGC AAA ACC TTG GCG ATG ATT TTT GAA AAA TCA TCG ACG CGG ACC CGC GTT TCG TTT GAG GCG GGC atg gcg

Figure S2. Hairpin-forming motifs and predicted structures of the WT and +/-Hairpin (120) strains. A. Hairpin structures predicted to form in the 120 bp region of each strain. B. Hairpin-forming motifs for each strain and the changes to the sequence (in red).

	WT	-Hairpin (40)
Mfold DNA fold	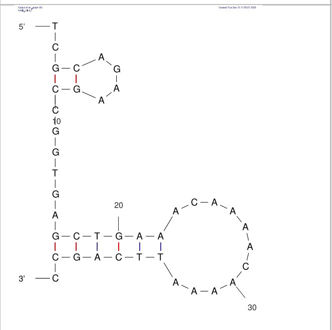	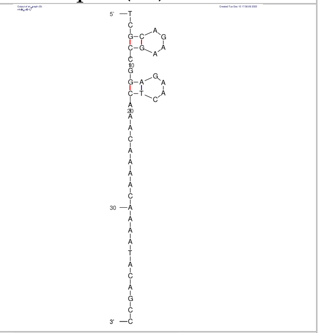
ΔG	-4.85 kcal/mol	-0.57 kcal/mol
Vienna DNA fold	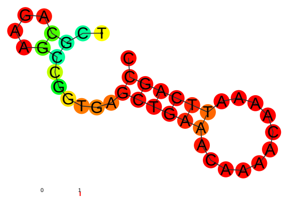	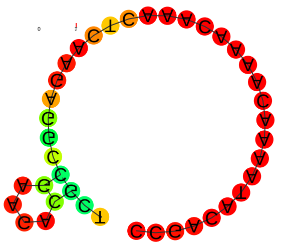
ΔG	-4.60 kcal/mol	-0.30 kcal/mol

WT Sequence starting at position 86-126 (40bp window)
cTC GCA GAA GCC GGT GAG CTG AAA CAA AAC AAA ATT CAG CCt
-Hairpin (40bp window)
cTC GCA GAA GCC GGA GAA CTC AAA CAA AAC AAA ATA CAG CCt

Figure S3. Hairpin-forming motif and predicted structure of WT and –Hairpin (40) strains. A. Hairpin structures predicted to form in the 40bp region of each strain. B. Hairpin-forming motifs for each strain and the changes to the sequence (in red).

2.1.2 Design of the +/-G4 strains

To design *argF* alleles differing in their ability to form G4 DNA, we initially used the web based tools: Quadruplex forming G-rich sequences (QGRS) mapper [11], ImGQfinder [12], Vienna RNAfold (DNA-folding quadruplexing-detecting parameter) [13], and G4-Predictor [14]. All of these tools have user-friendly web-based applications that allowed us to input the *argF* sequence and search for candidate G4 motifs. They predict G-quadruplex structures based on characteristics previously identified through thermodynamic studies to be important for stability such as: loop size [15], loop sequence [16], and the number of guanine tetrad planes [15]. Of note, ImGQfinder can identify "imperfect" G4 motifs with base mismatches and bulges [17][12][18]. In addition, QGRS mapper and ImGQfinder allow the user to change

some parameters including the number of guanines in a run, loop length, and motif length, while Vienna RNA-fold and G4-Predictor do not. This flexibility in parameters was reflected in our initial results, as Vienna RNA-fold and G4-Predictor did not find any candidate G4 motifs in *argF*. Contrastingly, QGRS Mapper found 7 using its most relaxed setting, and ImGQfinder found 15 using the most relaxed setting, which detected "imperfect" G4 motifs, but did not provide a stability score. Based on this results and analyses, we chose QGRS mapper as the tool of choice to design constructs with "perfect" G4 motifs with a sequence length <45 for this study.

We used QGRS mapper to search *argF* using different G4 motifs and found a few regions with potential to form G4 DNA structures with two tetrad planes with different loop lengths, we considered these good targets to introduce point mutations to increase stability. (Table S1). Interestingly, the same search across the *B. subtilis* coding regions in the genome did find G4 motifs with three or more guanine tetrad planes (Table S2). Similar to a recent study in *E. coli*, we found that the G4 motif $\mathbf{G_3}\mathrm{N}_{(1\text{-}5)}\mathbf{G_3}\mathrm{N}_{(1\text{-}5)}\mathbf{G_3}\mathrm{N}_{(1\text{-}5)}\mathbf{G_3}$ was not common in *B. subtilis* coding regions [19]. Additionally, searching with a flexible loop length drastically increased the number of G4 motifs found in *B. subtilis* coding regions (Table S2).

Table S1. QGRS Pattern search in argF ORF

Pattern searched	Count
GGG(1-5)GGG(1-5)GGG(1-5)GGG	0
GGG(1-7)GGG(1-7)GGG(1-7)GGG	0
GGG(1-11)GGG(1-11)GGG(1-11)GGG	0
GGG(1-36)GGG(1-36)GGG(1-36)GGG	0
GG(1-7)GG(1-7)GG(1-7)GG	2
GG(1-11)GG(1-11)GG(1-11)GG	2
GG(1-36)GG(1-36)GG(1-36)GG	7

Table S2. QGRS Pattern search in *B. subtilis* coding regions

Pattern searched	Count
**GGGG**(1-7)**GGGG**(1-7)**GGGG**(1-7)**GGGG**	0
**GGGG**(1-11)**GGGG**(1-11)**GGGG**(1-11)**GGGG**	0
**GGGG**(1-36)**GGGG**(1-36)**GGGG**(1-36)**GGGG**	4
**GGG**(1-5)**GGG**(1-5)**GGG**(1-5)**GGG**	1
**GGG**(1-7)**GGG**(1-7)**GGG**(1-7)**GGG**	4
**GGG**(1-11)**GGG**(1-11)**GGG**(1-11)**GGG**	25
**GGG**(1-13)**GGG**(1-13)**GGG**(1-13)**GGG**	39
**GGG**(1-36)**GGG**(1-36)**GGG**(1-36)**GGG**	590
**GG**(1-7)**GG**(1-7)**GG**(1-7)GG	4522

The most relaxed QGRS mapper motif search GG(1-36)GG(1-36)GG(1-36)GG with a maximum motif length of 45, found 7 sequences; these are unlike to form G4 because they do not have the four runs of three guanines (GGG)$_4$ (Table S3). We consider these good candidates to install GGG into the G4 alleles for our experiments. Here, all the G4 motifs detected in *argF* had at least two guanine tetrads and the predicted stability ranged from a G-score of 14 to 32 (Table S3).

Table S3. QGRS Results

Position	Length	G4 DNA Sequence	G-Score
198	**22**	GGGCATGGCGCAGCTGGGCGGG	**32**

282	**43**	**GGCAAAAGTGCTGTCAGGCTATGTGGATGCCATCAT GATCCGG**	**27**
342	**45**	**GGTGGAGGAACTTGCCAAAGAAGCTGACATTCCGGT GATCAATGG**	**15**
423	**42**	**GGATCTCTTGACGATTAAGGAGATAAAAGGGAAGCT TAAAGG**	**29**
484	**38**	**GGTGACGGAAATAACGTGGCGCACTCCTTGATGATC GG**	**23**
705	**14**	**GGGGCAGGAAGCGG**	**32**
843	**44**	**GGAGATTATTGACGGGCCGAACTCTGCAGTGTTCCA GCAGGCGG**	**14**

We chose the G4 motif that started at position 198 and had a G-score of 32 for our study. To create the +G4 strain and increase the stability of the endogenous G4 in *argF*, we introduced 3 synonymous point mutations in the 36 bp sequence that resulted in a G4 motif change from $\mathbf{G_2}N_{(1-7)}\mathbf{G_2}N_{(1-7)}\mathbf{G_2}N_{(1-7)}\mathbf{G_2}$ to $\mathbf{G_3}N_{(1-13)}\mathbf{G_3}N_{(1-13)}\mathbf{G_3}N_{(1-13)}\mathbf{G_3}$ (Figure S4). While the G4 motif used in this study deviates from the most widely used one, which has three guanine tetrads and a fixed loop length for all three loops of 7, $\mathbf{G_3}N_7\mathbf{G_3}N_7\mathbf{G_3}N_7\mathbf{G_3}$, this new motif was demonstrated to generate a G4 tetrad [15]. QGRS mapper predicted that the sequence changes added an additional tetrad plane and resulted in an increased G-score (G-score 60). To create the -G4 strain, we introduced seven-point mutations to the 36 bp sequence, which effectively disrupted all guanine runs in the region, resulting in a G-score of 0. Of note, 2 of the 7-point mutations were not synonymous and resulted in a glycine to alanine change at residues 67 and 72. Although glycine and alanine are similar in properties, we confirmed these changes did not result in any growth defects when compared to the WT *argF*.

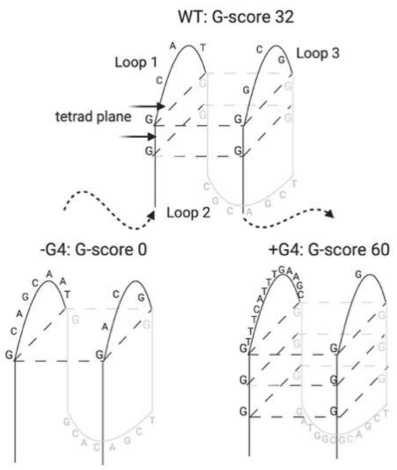

	Sequence	pattern
**WT**	GT GTT TCA TTT GAA GCG GGC ATG GCG CAG CTG GGC GGG	$\mathbf{G_2}N_4\mathbf{G_2}N_7\mathbf{G_2}N_3\mathbf{G_2}$
**+G4**	GG GTT TCA TTT GAA GCG GGG ATG GCG CAG CTG GGG GGG	$\mathbf{G_3}N_{13}\mathbf{G_3}N_{12}\mathbf{G_3}N_1\mathbf{G_3}$
**-G4**	GT GTT TCA TTT GAA GCA GCA ATG GCA CAG CTG GCA GGC	$\mathbf{G_1}N_7\mathbf{G_1}N_8\mathbf{G_1}N_3$ **G1**

Figure S4. G4-forming motif and predicted structure of +G4 and -G4 strains. Schematic of G4 DNA structure predicted to form in the 36bp sequence of each strain and the G4-forming motifs for each strain. Changes to the sequence are shown in red, and the overall pattern is also depicted.

2.1.3 Codon analysis

We further analyzed the new *argF* alleles containing stable or unstable non-B DNA sequences for indirect effects on DNA structure and changes in codon bias. Although *B. subtilis* does not exhibit a strong codon bias[20][21], we considered the effect of the engineered changes into the different constructs on codon bias and as potential confounding factor in our experiments. Using publicly available codon usage table for *B. subtilis* from the Kazusa Codon usage database, we compared the frequencies of codons, per residue position, in the WT and those found in each of the constructs. On average across the protein-coding region, we found no significant changes in codon usage between the WT and the engineered constructs (Supplementary Figure S5).

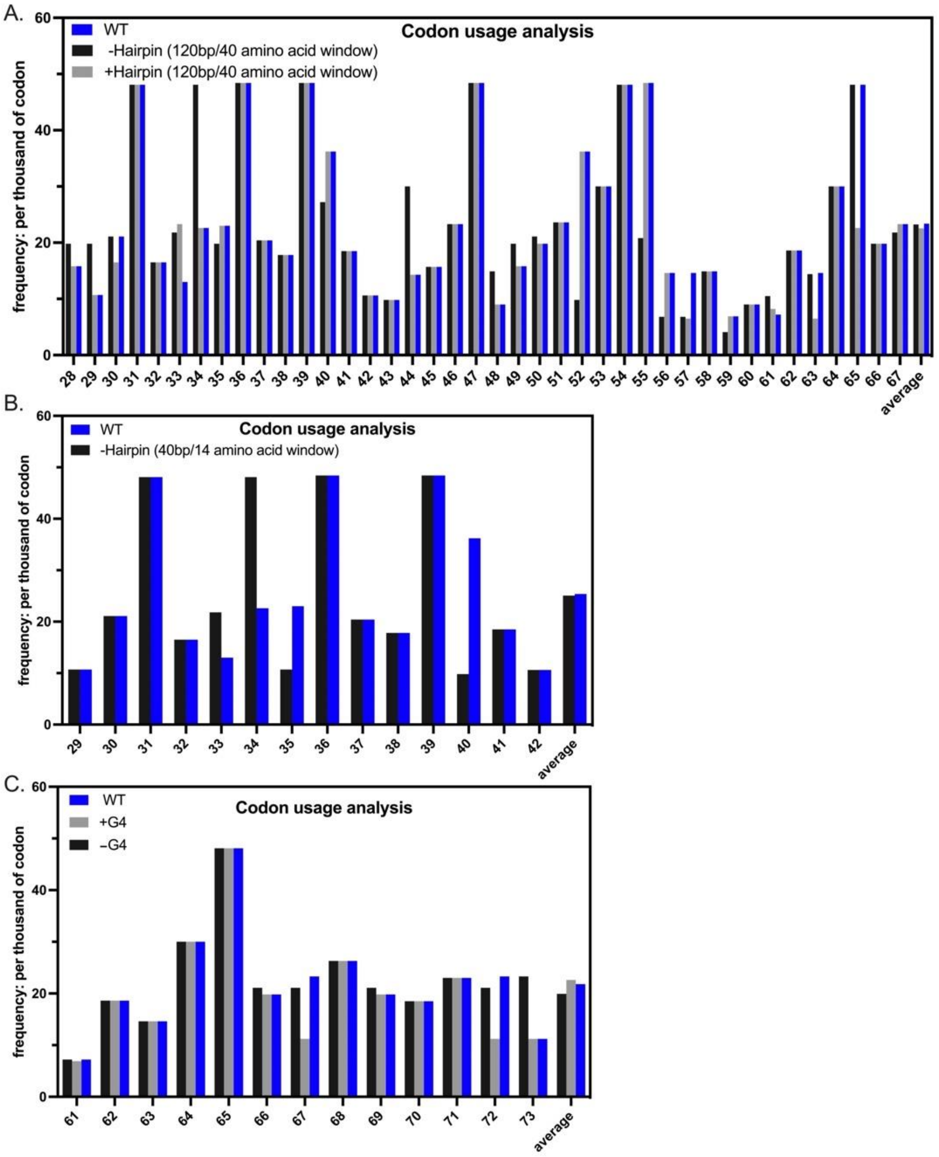

Figure S5. Codon analysis using the Kazusa Codon Usage Database of regions in *argF* that were changed to disrupt or stabilize an endogenous non-B DNA structure. The frequency: per thousand was plotted for every codon that spanned the non-B DNA-forming motif. The average frequency: per thousand across the region was also calculated and plotted as the average A. Codon analysis of +/-Hairpin (120) strains compared to the WT. B. Codon analysis of -Hairpin (40) strain compared to the WT. C. Codon analysis of +/-G4 strains compared to the WT.

2.1.4 Addition of stop codon

Once the *argF* alleles were designed, we introduced a nonsense codon to the sequence at a position corresponding to the loop region of the structure, which resulted in a non-functional truncated ArgF and a system to measure reversions to Arg^+. The type of nonsense codon and placement for each strain set was chosen based on three factors: 1) sequence limitations, what codons could be changed with a 1 b point mutation into a nonsense codon; 2) location within the structure, the loop regions are expected to be exposed and prone to lesions; 3) residue conservation, we targeted codons of amino acid residues that are not highly conserved to facilitate the production of broader spectrum of mutations conferring prototrophy.

Based on these criteria, we introduced an ochre nonsense codon at position 37Q (CAA→TAA) corresponding to the loop part of the predicted hairpin before disruption. For the +/-G4 strains, we introduced an amber nonsense codon at position 70Q (CAG→TAG) corresponding to the second loop of the predicted G4 structure. Of note, the WT *argF* gene contains an opal stop codon TGA. Additionally, in *B. subtilis* and other bacteria belonging to the Firmicutes phyla, TAA is predominantly used as a translational stop over TAG [22].

2.1.5 Cloning and construction of the non-B DNA strains

Once the strains were designed with the addition of the nonsense codon *in silico*, the new *argF* sequences containing the changes to the ORF were sent to Gene Universal (Newark, DE) for synthesis. We added restriction site sequences to the beginning and the end of *argF* allele sequences to allow for downstream cloning steps. The primers *ArgF WT fwd SalI* and *ArgF WT rvs SphI* denote the start and end of the *argF* alleles along with the restriction site sequences added (Table S4). The synthesized constructs were cloned into the cloning vector pUC57. The pUC57 derivatives were digested with NheI and SalI following standard reaction protocol (New England Biolabs, Ipswich, MA). After digestion, the products were resolved on a low melting 1% agarose gel and fragments that corresponded to the ~1056 size were excised out and cleaned up using Qiagen MinElute Gel Extraction Kit (Venlo, Netherlands). The DNA fragment was ligated to pDR111 which was also digested with the same restriction enzymes. The vector pDR111 contains an IPTG-inducible promoter and has fragments with homology to the *amyE* region of *B. subtilis* chromosome to promote recombination (Rudner lab, Harvard). The ligated plasmid was transformed into competent *B. subtilis cells* with the native *argF* deleted (CV4000) [4] and selected using complex medium (TBAB) with 100 μg/ml of spectinomycin and 100 μg/ml of neomycin. Strains were confirmed via phenotype (See table S5) and PCR sequencing using Pspac F primer and ArgF WT Rvs. The Mfd^- strains were constructed by transforming genomic DNA from YB9801 [23] into the +/-hairpin and +/-G4 strains and selecting on TBAB plates containing tetracycline.

Table S4. Primers used in this study

**Primer**	**Sequence**
argF WT FWD SalI	5' GAT GTCGAC TAAAAAGGAAGTGGCATC ATG CAC ACA GTG ACG CAA 3'
argF WT RVS SphI	5' ACCA GCATGC CC TCC TTT TCC TTT TTG CTG TAG TAT GC 3'
Pspac F	5' ACT TTA TCT ACA AGG TGT GGC ATA ATG TGT GGA A-3'

Table S5. Summary of Phenotype Test

	**TBAB**	**Minimal Media lacking arginine**	**Minimal Media lacking arginine +.1mM IPTG**	**Minimal Media +100ug/ml Arginine**
**YB955**[24]	+	+	+	+
arg::neo[4]	+	-	-	+
**+Hairpin**	+	-	-	+
**-Hairpin**	+	-	-	+
**+G4**	+	-	-	+
**-G4**	+	-	-	+

Each strain was grown overnight, washed to remove excess nutrients, and spotted on different media types. Growth was assessed after 48hrs for growth (+) or no growth (-).

3.1. *Non-B DNA*-forming motifs *promote mutagenesis*

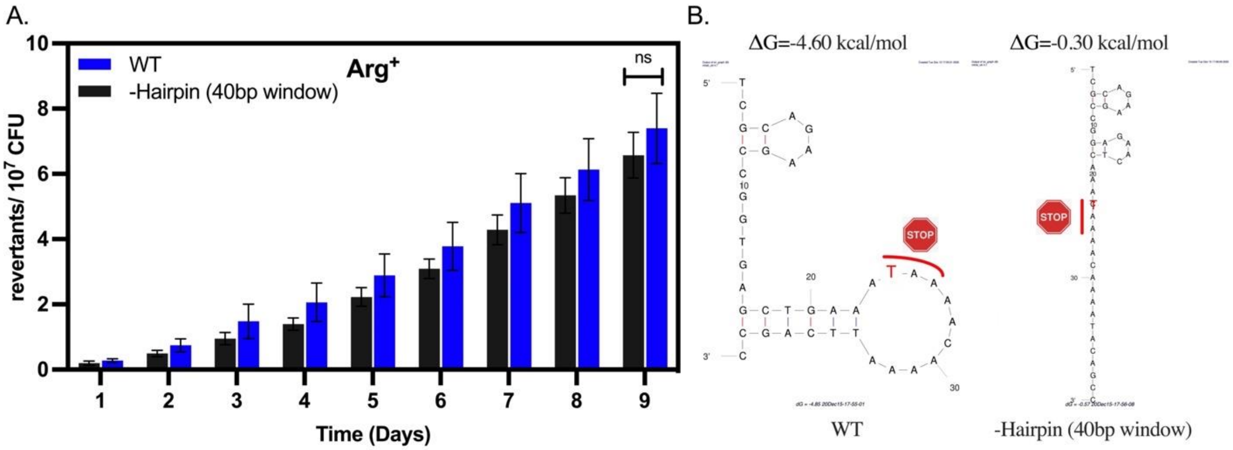

Figure S6. The -Hairpin (40bp strain) is indistinguishable from the W.T. A. Stationary-phase mutagenesis assay results for -Hairpin (40) strain compared to WT. B. Mfold predicted hairpin structures for WT and the -Hairpin 40bp region in *argF* (positions 86-126).

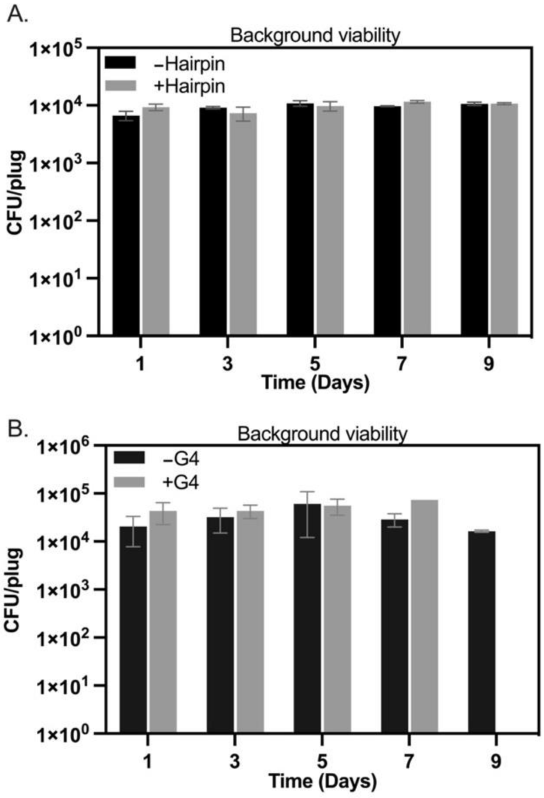

Figure S7. Viability of the non-revertant population for the non-B DNA strains during the SPM assay. Of note, we were unable to assess survival of the +G4 strain after day 7 because the plates became too crowded with Arg⁺ mutants to take an agar plug.

TAG vs TAA

We did notice that the +/-G4 strains accumulated more mutants over time compared to the +/- hairpin strains. This is consistent with our previous observations [25], and we suspect that the difference in mutagenesis is based on the intrinsic differences between the type of nonsense codon each strain set contained. Here we explore those differences briefly and their potential impact on mutagenesis levels; we consider sequence context, tRNA suppression, and efficiency of translation termination to explain the differences observed in this study.

Two types of mutants can lead to Arg^+ in our system, *argF* mutants and a nonsense suppressor tRNA, and both were higher in the +/-G4 strains compared to the +/- hairpin strains. This contributed to an overall higher mutation frequency in stationary phase and different y-scales in figures 2 and 5. Sequence analysis revealed that the +/-G4 strains acquired mutations in the 1st and 3rd position of the amber nonsense codon (TAG) in the *argF* gene, while we only detected mutations in the 1st position of the ochre nonsense codon (TAA) in the +/-Hairpin strains. The T→C transition was common for the first position of both nonsense codons, but G→T transversions were observed only in the 3rd position of the amber nonsense codon (TAG; -/+ G4 strains). These data suggest that the TAG stop codon is more permissive of mutations that confer prototrophy.

For the suppressor mutations, we speculate that nonsense tRNA suppressors were increased in the +/-G4 strains containing the amber TAG stop because this stop codon can be suppressed by both amber suppressor tRNAs and ochre suppressor tRNAs; the ochre nonsense codon is suppressed by ochre suppressor tRNAs only [26]. Therefore, there are more tRNA's that can mutate into amber suppressors than ochre suppressors; this leads to increased nonsense tRNA suppressors and higher Arg^+ levels in the G4 strains.

We also consider termination efficiency. The ochre nonsense codon (TAA, - Hairpin strains) has more efficient translation termination because it is recognized by both translation release factors RF1 and RF2; the amber nonsense codon (TAG) is only recognized by one [22]. Studies have shown that the inhibition of translation decreases transcription efficiency [27], and we demonstrated that increased transcription leads to increases in mutagenesis in stationary phase [28]. Perhaps, the inefficient translation termination in the +/-G4 strains facilitates increases in transcription efficiency and increases in Arg^+ mutagenesis.

In summary, it is most likely, the intrinsic differences between the stop codons used in the Hairpin and G4 strains contributed to the differences in stationary-phase mutagenesis levels between the two sets of strains. However, our assays, within each set of strains, showed that sequences with non-B DNA motifs have a significant effect on mutagenesis in stressed cells.

3.3. Mfd promotes mutations at non-B DNA sequences

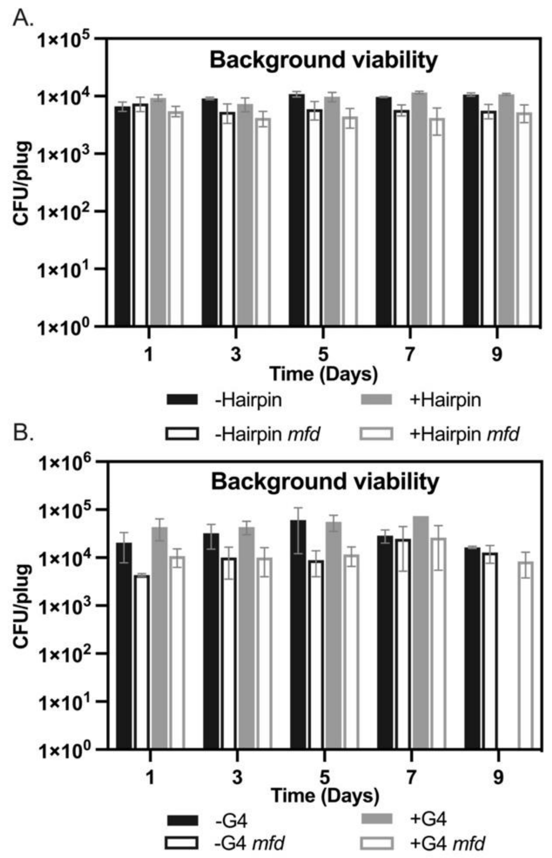

Figure S8. Viability of the non-revertant population for the non-B DNA strains and their Mfd deficient counterpart during the SPM assay. Of note, we were unable to assess survival of the +G4 strain after day 7 because the plates became too crowded with Arg$^+$ mutants to take an agar plug.

4.1 An in vivo system to measure the effects of non-B DNA-forming motifs on bacterial mutagenesis

A.

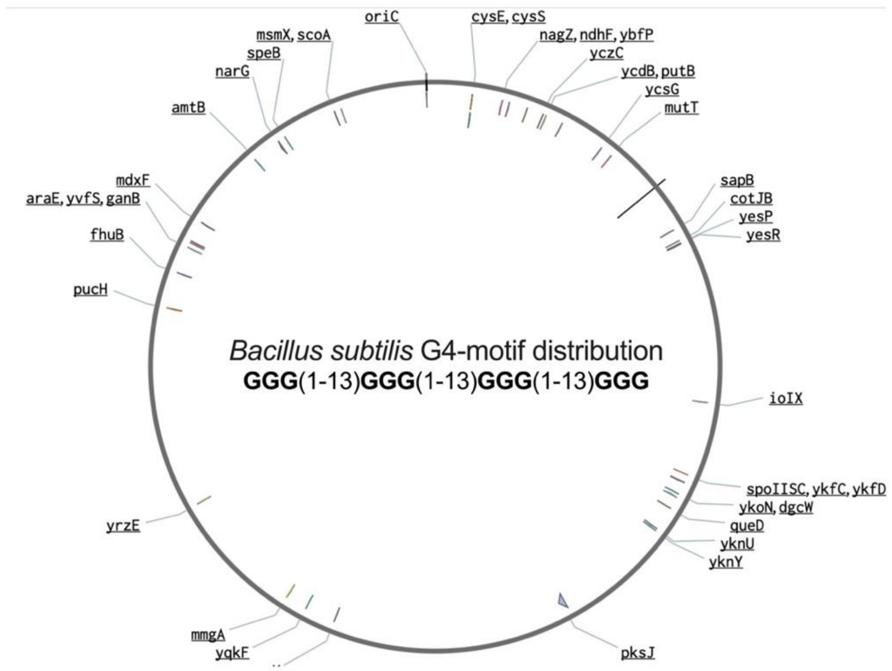

B.

gene	description	length	G4 motif	QGRS score
cysE	serine O-acetyltransferase	35	GGGGTTACCCTCGGGGGAACGGGAAAAGAAAAGGG	68
cysS	dual cysteinyl-tRNA synthetase; cysteine persulfide synthase	31	GGGATAGCCCTTGGGGGAAAGGGCGTCCGGG	68
nagZ	β-N-acetyl-glucosaminidase	38	GGGGAGGGCACTAATTTCCCAGGGAATATGGCGCTGGG	61
ndhF	putative NADH dehydrogenase; prophage 1 region	27	GGGCTGGGAGAATGGGAGAGAACAGGG	66
ybfP	putative transcriptional regulator (AraC/XylS family)	43	GGGGATTGACCGGGAAAATGTACATCAGGGAAAAGCTTCAGGG	67
yczC	putative integral membrane protein of unknown function	44	GGGCACACTGGGCAAAAAAATCATCGGGCTGCAAATCGTGAGGG	65
ycdB	conserved protein of unknown function	39	GGGTTTGTCAAAGAGCGGGAAGCGGATTTGGGAGATGGG	63
putB	proline oxidase	38	GGGGAAGTCGGGTAGCAGCGGGTAAAATTATCGGCGGG	65
ycsG	putative transporter YcsG	26	GGGAATATCGGAGGGGCCGGGCTGGG	65
mutT	putative NTP pyrophosphohydrolase	32	GGGATTTGCCGGGCGGCAGGGTTGATCCCGGG	69
sapB	putative magnesium transporter	30	GGGGGCAGCGGGGCTCGGGCTTGCTACGGG	68
cotJB	component of the inner spore coat	28	GGGCAAGGATTGGGATTGGGGAAAAGGG	68
yesP	putative carbohydrate ABC transporter membrane subunit YesP	25	GGGCGAGGGCGGGCCGCTTGACGGG	64
yesR	rhamnogalacturonan hydrolase	31	GGGATTTATTGGGGCAGGGCGAACGGCTGGG	66
iolX	scyllo-inositol 2-dehydrogenase (NAD(+))	41	GGGCGGAGGTGATCGGGACAGAGGGCAGTATTTTCATAGGG	64
spoIISC	three component toxin / antitoxin / antitoxin SpoIISABC, antitoxin C	31	GGGCTTTCCGGGTACTCGCTTCGGGTGAGGG	65
ykfC	gamma-D-glutamyl-L-diaminoacid endopeptidase	23	GGGGAGGGATCAGCGGGTTTGGG	68
ykfD	ABC transporter ATP-binding proteinYkfD	27	GGGCTAGTCGGGGAATCAGGGTGCGGG	69
ykoN	putative glycosyltransferase	36	GGGGCAGTATGGGGGTCGGTGGGATTTTAAAATGGG	69
dgcW	diguanylate cyclase	34	GGGCATTGGGATTTCGGGCATGCACTACACAGGG	63
queD	6-carboxy-5,6,7,8-tetrahydropterin synthase; queuosine biosynthesis	35	GGGAAAGTGCGGGAGCGTGCAGCAGGGTTCACGGG	66
yknU	ABC family transporter ATP-binding/membrane protein YknU	31	GGGAGAGAGGGGAATGGGGCTTTCCGGCGGG	68
yknY	putative transporter (SDP resistance) ATP-binding protein	35	GGGCGGGCAGAAGCAGCGGGTGGCGATTGCGAGGG	61
pksJ	polyketide synthase of type I involved in nonribosomal synthesis of bacillaene	33	GGGCAAAAGAACAAGGGCTTGAAGGGTTCAGGG	65
yqkF	NADPH-dependent aldo-keto reductase (acts on 4-hydroxy-2,3-trans-nonenal)	41	GGGAAACCGTTGGGATGACGGAAGCGAGGGCTGGTATTGGG	67
mmgA	degradative acetoacetyl-CoA thiolase	40	GGGCGCTGGGGAGCACGGATGGGGGACGGCGAGCTTAGGG	65
yrzE	putative transporter	31	GGGATTTTTAGGGGTGTGCATGCTGGGCGGG	63
menH	demethylmenaquinone methyltransferase	43	GGGCTCAAAACACTTGGGGAACGGCGGGAACGAATCATGCGGG	67
pucH	allantoinase	21	GGGCAGGGAGGATTGGGAGGG	67
fhuB	iron(III) hydroxamate ABC transporter membrane subunit FhuB	33	GGGAGCGGGGCTGGGGGCATCGACGGTAATGGG	63
araE	arabinose-related compounds permease	22	GGGGGCTGGGAATCGGGATGGG	70
yvfS	putative ABC efflux transporter membrane protein YvfS	33	GGGAGCGGAGCGTGGGAACTTGTCCGGGGTGGG	64
ganB	arabinogalactan endo-1,4-β-galactosidase	24	GGGAAACATACGGGTCAGGGTGGG	65
mdxF	maltodextrin ABC transporter membrane subunit MdxF	28	GGGAGGGCCAGGCAGTGCGGGAGCCGGG	62
amtB	ammonium transporter	44	GGGCTGGAGTGGGCAGGCCTCAAAGGGGTCGGATTTGATCCGGG	66
narG	nitrate reductase (alpha subunit)	27	GGGACAATGGGCTCGCGCTGGGACGGG	66
speB	agmatinase	27	GGGAAGTTCCCAATGGGTATGGGCGGG	62
msmX	multiple sugar ABC transporter ATP-binding protein	30	GGGTTGCGCTGGGCCGGGCAATCGTGCGGG	65
scoA	acetoacetyl CoA-transferase (subunit A)	29	GGGATACGCTGATCGCGGGAGGGTTTGGG	60

Figure S9. A. Schematic showing the chromosomal distribution of the 39 genes that contained the G4 motif used in this study. B. Table with the gene name and description of the 39 genes that contained the G4 motif used in this study, along with the motif sequence and QGRS score.

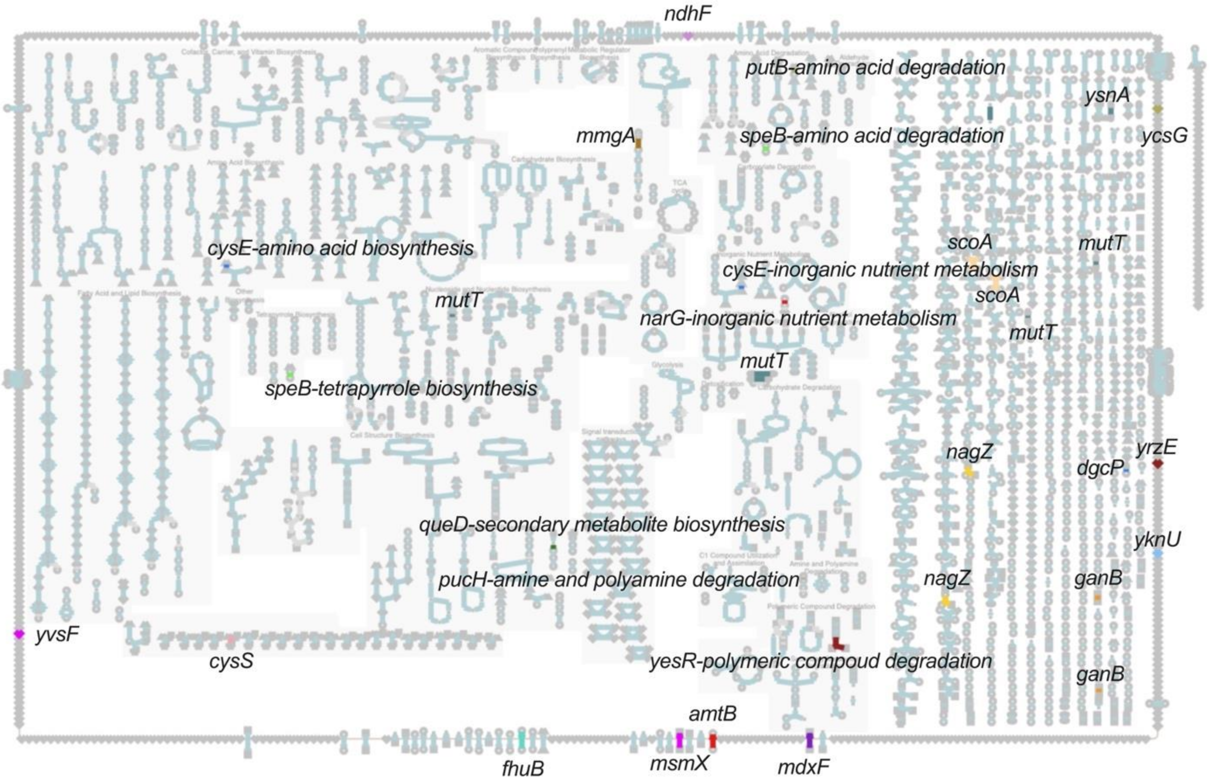

Figure S10. Modified figure from Bsucyc.org showing the cellular function of the 39 genes that contained the G4 motif used in this study.

References

[1] R. Cunin, N. Glansdorff, A. Piérard, and V. Stalon, “Biosynthesis and metabolism of arginine in bacteria.,” *Microbiol. Rev.*, vol. 50, no. 3, pp. 314–352, 1986.

[2] M. O. Reilly and K. M. Devine, “Sequence and analysis of the citrulline biosynthetic operon argC-F from Bacillus subtilis,” *Microbiology*, no. 140, pp. 1023–1025, 1994.

[3] K. Makarove, A. A. Mironov, M. S. Gelfand, and K. S. Makarova, “Conservation of the binding site for the arginine repressor in all bacterial lineages,” *Genome Biol.*, vol. 2, no. 4, p. RESEARCH0013, 2001.

[4] H. A. Martin *et al.*, “Mfd protects against oxidative stress in Bacillus subtilis independently of its canonical function in DNA repair,” *BMC Microbiol.*, vol. 19, no. 26, pp. 1–14, 2019.

[5] M. Zuker, “Mfold web server for nucleic acid folding and hybridization prediction,” *Nucleic Acids Res.*, vol. 31, no. 13, pp. 3406–3415, 2003.

[6] I. L. Hofacker, “Vienna RNA secondary structure server,” *Nucleic Acids Res.*, vol. 31, no. 13, pp. 3429–3431, 2003.

[7] K. H. Schmidt, J. M. Reimers, and B. E. Wright, “The effect of promoter strength, supercoiling and secondary structure on mutation rates in Escherichia coli,” *Mol. Microbiol.*, vol. 60, no. 5, pp. 1251–1261, 2006.

[8] A. Naidoo, K. Naidoo, N. Yende-zuma, and T. N. Gengiah, “Correlations between secondary structure stability and mutation frequency during somatic hypermutatio,” *Mol. Immunol.*, vol. 19, no. 2, pp. 161–169, 2008.

[9] K. Szlachta, R. G. Thys, N. D. Atkin, L. C. T. Pierce, S. Bekiranov, and Y.-H. Wang, “Alternative

DNA secondary structure formation affects RNA polymerase II promoter-proximal pausing in human," *Genome Biol.*, vol. 19, no. 1, p. 89, 2018.
[10] N. R. Markham and M. Zuker, "DINAMelt web server for nucleic acid melting prediction," *Nucleic Acids Res.*, vol. 33, no. Web Server, pp. W577–W581, 2005.
[11] O. Kikin, L. D. Antonio, and P. S. Bagga, "QGRS Mapper : a web-based server for predicting G-quadruplexes in nucleotide sequences," *Nucleic Acids Res.*, vol. 34, no. 21, pp. 676–682, 2006.
[12] A. Varizhuk *et al.*, "An Improved Search Algorithm to Find G-Quadruplexes in Genome Sequences," *bioRxiv*, 2014.
[13] V. Package, R. Lorenz, S. H. Bernhart, C. Höner, H. Tafer, and C. Flamm, "ViennaRNA Package 2.0," *algorithms Mol. Biol.*, pp. 1–14, 2011.
[14] S. K. Mishra, A. Tawani, A. Mishra, and A. Kumar, "G4IPDB : A database for G-quadruplex structure forming nucleic acid interacting proteins," *Nat. Publ. Gr.*, no. December, pp. 2–10, 2016.
[15] A. Guédin, J. Gros, P. Alberti, and J. L. Mergny, "How long is too long? Effects of loop size on G-quadruplex stability," *Nucleic Acids Res.*, vol. 38, no. 21, pp. 7858–7868, 2010.
[16] A. Guédin, P. Alberti, and J. L. Mergny, "Stability of intramolecular quadruplexes: Sequence effects in the central loop," *Nucleic Acids Res.*, vol. 37, no. 16, pp. 5559–5567, 2009.
[17] V. T. Mukundan, "Bulges in G-Quadruplexes: Broadening the Defi nition of G-Quadruplex-Forming Sequences," 2013.
[18] A. Varizhuk, D. Ischenko, V. Tsvetkov, and R. Novikov, "The expanding repertoire of G4 DNA structures Biochimie The expanding repertoire of G4 DNA structures," *Biochimie*, no. January, 2017.
[19] T. Endoh, Y. Kawasaki, and N. Sugimoto, "Suppression of Gene Expression by G-Quadruplexes in Open Reading Frames Depends on G-Quadruplex Stability," *Angew Chem Int Ed Engl*, pp. 5522–5526, 2013.
[20] N. Ogasawara, "Markedly unbiased codon usage in Bacillus subtiiis," *Gene*, vol. 40, pp. 145–150, 1985.
[21] G. W. Li, E. Oh, and J. S. Weissman, "The anti-Shine-Dalgarno sequence drives translational pausing and codon choice in bacteria," *Nature*, vol. 484, no. 7395, pp. 538–541, 2012.
[22] G. Korkmaz, M. Holm, T. Wiens, and S. Sanyal, "Comprehensive Analysis of Stop Codon Usage in Bacteria and Its Correlation with Release Factor Abundance," *J. Biol. Chem.*, vol. 289, no. 44, pp. 30334–30342, 2014.
[23] C. Ross, C. Pybus, M. Pedraza-Reyes, H. M. Sung, R. E. Yasbin, and E. Robleto, "Novel role of mfd: Effects on stationary-phase mutagenesis in Bacillus subtilis," *J. Bacteriol.*, vol. 188, no. 21, pp. 7512–7520, 2006.
[24] H.-M. Sung and R. E. Yasbin, "Adaptive, or stationary-phase, mutagenesis, a component of bacterial differentiation in Bacillus subtilis," *J. Bacteriol.*, vol. 184, no. 20, pp. 5641–5653, 2002.
[25] H. M. Sung and R. E. Yasbin, "Adaptive, or stationary-phase, mutagenesis, a component of bacterial differentiation in Bacillus subtilis," *J. Bacteriol.*, vol. 184, no. 20, pp. 5641–5653, 2002.
[26] G. Eggertsson and D. Soll, "Transfer Ribonucleic Acid-Mediated Suppression of Termination Codons in Escherichia coli," *Microbiol. Mol. Biol. Rev.*, vol. 52, no. 3, pp. 354–374, 1988.
[27] K. McGary and E. Nudler, "RNA polymerase and the ribosome: The close relationship," *Curr. Opin. Microbiol.*, vol. 16, no. 2, pp. 112–117, 2013.
[28] C. Pybus *et al.*, "Transcription-Associated Mutation in Bacillus subtilis Cells under Stress," *J. Bacteriol.*, vol. 192, no. 13, pp. 3321–3328, 2010.
